# Wnt7b Inhibits Osteoclastogenesis *via* AKT Activation and Glucose Metabolic Rewiring

**DOI:** 10.3389/fcell.2021.771336

**Published:** 2021-11-22

**Authors:** Fanzi Wu, Boer Li, Xuchen Hu, Fanyuan Yu, Yu Shi, Ling Ye

**Affiliations:** ^1^State Key Laboratory of Oral Diseases, National Clinical Research Center for Oral Diseases, West China Hospital of Stomatology, Sichuan University, Chengdu, China; ^2^Department of Endodontics, West China Hospital of Stomatology, Sichuan University, Chengdu, China

**Keywords:** Wnt7b, osteoclastogenesis, AKT, glucose metabolism, osteoporosis

## Abstract

The imbalance between bone formation and bone resorption causes osteoporosis, which leads to severe bone fractures. It is known that increases in osteoclast numbers and activities are the main reasons for increasing bone resorption. Although extensive studies have investigated the regulation of osteoclastogenesis of bone marrow macrophages (BMMs), new pharmacological avenues still need to be unveiled for clinical purpose. Wnt ligands have been widely demonstrated as stimulators of bone formation; however, the inhibitory effect of the Wnt pathway in osteoclastogenesis is largely unknown. Here, we demonstrate that Wnt7b, a potent Wnt ligand that enhances bone formation and increases bone mass, also abolishes osteoclastogenesis *in vitro*. Importantly, enforced expression of Wnt in bone marrow macrophage lineage cells significantly disrupts osteoclast formation and activity, which leads to a dramatic increase in bone mass. Mechanistically, Wnt7b impacts the glucose metabolic process and AKT activation during osteoclastogenesis. Thus, we demonstrate that Wnt7b diminishes osteoclast formation, which will be beneficial for osteoporosis therapy in the future.

## Introduction

Osteoporosis is an emerging global epidemic that severely increases the life burden of patients. It is well known that the imbalance between osteoblast-mediated bone formation and osteoclast-mediated bone resorption causes osteoporosis (Lei et al., 2006; Hernlund et al., 2013). Recently, increasing studies have focused on the regulation of osteoblasts and that of osteoclasts. Osteoclasts are primarily bone-resorption cells and play an important role in bone homeostasis (Udagawa et al., 1990). Multinucleated giant cells are formed by the fusion of myeloid hematopoietic precursors and attach to bone surfaces to perform bone resorption (Yasuda et al., 1998). Multiple factors are involved in osteoclastogenesis and osteoclast activation. Indeed, hormones, cytokines, nutrients, and inflammatory factors can positively or negatively regulate osteoclast formation, most importantly RANK ligand (RANKL), which drives osteoclast precursors to differentiate into TRAP-positive multiple nucleated cells *via* numerous signaling pathways (Teitelbaum and Ross, 2003). RANKL is secreted by surrounding osteoblast-lineage cells, which provide a suitable microenvironment for osteoclast differentiation (Yasuda et al., 1998; Teitelbaum and Ross, 2003; Liu and Zhang, 2015).

Wnt signaling has emerged as a promising pathway in skeletal development (Richards et al., 2008; van Amerongen and Nusse, 2009; Maupin et al., 2013). Several Wnt ligands have been identified as potential drugs for osteogenesis imperfecta and fracture healing (Baron and Gori, 2018). The two well-known Wnt pathways are the β-catenin-dependent pathway (also known as the canonical Wnt pathway) and the β-catenin-independent pathway (also known as the non-canonical Wnt pathway) (Huybrechts et al., 2020). The canonical Wnt pathway transduces signals through LRP/Frz receptors on the cell surface. In contrast, non-canonical Wnt signaling modulates cell fate commitment and differentiation through binding to Ror1 and Ror2 (Hovanes et al., 2001; Kimelman and Xu, 2006; Tu et al., 2007). It has been reported that both canonical and non-canonical Wnt pathways are crucial during osteoblast formation (Day et al., 2005; Tu et al., 2007). Loss-of-function mutations of LRP5 signals have been shown to cause osteoporosis by reducing the number of osteoblasts. Moreover, mice with gain-of-function mutations of LRP5 exhibited high bone mass phenotype (Cui et al., 2011). Wnt5a, a typical non-canonical Wnt ligand, has also been reported to be involved in bone formation. Specific Wnt5a deletion in mice osteoblasts caused a low bone mass phenotype with deceased osteoblast numbers and a reduced bone formation rate (Maeda et al., 2019). Considering that osteoblasts express osteoprotegerin (OPG) to block the interaction between RANKL and RANK, Wnt signals play a pivotal role in osteoclast formation. Although current studies have also focused on the direct modulatory role of Wnt signaling in osteoclast differentiation, the results remain contradictory. Constitutively active β-catenin in osteoclast precursors barely forms osteoclasts, whereas the β-catenin signal is required for the proliferation of osteoclast precursors (Sui et al., 2018). Furthermore, Wnt5a has been shown to activate the Ror2-JNK signaling and enhance RANKL-induced osteoclastogenesis (Maeda et al., 2012). On the contrary, Wnt16 and Wnt4 impair osteoclast formation by blocking the RANKL–RANK axis (Movérare-Skrtic et al., 2014; Yu et al., 2014).

Among Wnt ligands, Wnt7b has a profound effect on bone formation. Recent evidence indicates that Wnt7b contributes to the commitment and differentiation of the osteoblast lineage through multiple signaling pathways and rewiring of metabolic processing to enhance osteogenic function (Chen et al., 2014, 2019; Yu et al., 2020). Our group observed that forced activation of Wnt7b in mature osteoblasts *in vivo* (taking advantage of *Wnt7b^*DMP*1^*mice) leads to a high bone mass phenotype. In detail, we demonstrate that Wnt7b enhanced self-renewal and osteogenic differentiation of bone marrow stromal cells (BMSCs) through Sox11 (Yu et al., 2020). Surprisingly, we also observed that osteoclasts are decreased in *Wnt7b^*DMP*1^* mice. These data lead us to hypothesize that Wnt7b plays a negative role in osteoclast differentiation. However, the direct effect of Wnt7b in osteoclast formation remains underexplored.

In this study, we generate transgenic mice in which Wnt7b is specifically activated in BMMs to uncover an inhibitory role of Wnt7b during osteoclast formation and activation. We also further demonstrate that Wnt7b inhibits the differentiation of osteoclast precursors by disrupting AKT phosphorylation and rewiring glucose metabolism.

## Materials and Methods

### Mouse Strain

ROSA26-Wnt7b^flox/flox^ mice were provided by Prof. Fanxin Long from University of Pennsylvania. LysM-Cre or RANK-Cre were purchased from Biocytogen Co. Ltd., Beijing, China. All animal procedures were approved by the Ethical Committees of the State Key Laboratory of Oral Diseases, Sichuan University. The research was carried out in accordance with accredited guidelines. Genotype was identified by PCR analysis with primers for ROSA26-Wnt7b^flox/flox^, LysM-Cre, and RANK-Cre.

### Micro-CT Analysis

All procedures were performed according to reported methods. The right femurs were isolated, fixed in 4% paraformaldehyde, and maintained in phosphate-buffered saline (PBS) containing 0.4% paraformaldehyde. All radiographic data were acquired *via* VIVA 40CT 64GB (Scanco Medical AG, Bassersdorf, Switzerland) system using previously established parameters as follows: X-ray tube potential, 55 kVp; X-ray intensity, 145 μA; integration time, 200 ms; and threshold, 220 mg/cm^3^. The percentage of bone volume (BV/TV), trabecular thickness (tb.Th.), trabecular number (tb.N.), trabecular separation (tb.Sp), and cortical thickness (ct,Th.) were conducted, and 3D images were reconstructed for visualization.

### Bone Dynamic Analysis

Eight-week-old mice were labeled with calcein (10 mg/kg, sigma) and alizarin red (30 mg/kg, Sigma) through intraperitoneal injection at 5 and 3 days before sacrificed. Undecalcified femurs were fixed with 4% paraformaldehyde and dehydrated with 30% sucrose dissolved in PBS. Twenty-micrometer sections were performed for microscopy analysis. Images were captured under 510–550 nm and 450–480 nm fluorescent light to image calcein and alizarin. Image processing was used Image Pro Plus software.

### Detection of Serum Biomarkers

Mice 6 and 8 weeks old were fasted for at least 6 h; blood was drawn, allowed to clot at room temperature for 30 min, and then centrifuged at 3,000 rpm for 10 min at 4°C. Serum samples were stored at −80°C. ELISA was employed to determine mouse serum PINP and CTX-I (cloud clone, Wuhan, China) according to the manufacturer’s protocol. Data processing was performed with Curve expect software.

### Cell Culture

Primary bone marrow macrophages (BMMs) were obtained from femurs or tibias of 6–8-week-old C57BL6/J male mice, according to a published method. The cells were cultured and expanded with α-MEM (M0644, Sigma, United States) containing 10% FBS, 100 U/mL penicillin, 100 μg/mL streptomycin, and 50 ng/mL macrophage colony-stimulating factor (M-CSF) for 4 days (Li et al., 2020). For osteoclast differentiation, the adherent BMMs were seeded at 3.125 × 10^4^ cells/cm^2^ and induced with 20 ng/mL M-CSF and 50 ng/mL RANKL for 3–5 days. RAW 264.7 were cultured in the same conditions without M-CSF, and 50 ng/mL RANKL was sufficient for osteoclast differentiation induction. For TRAP staining, cells were fixed with 4% of paraformaldehyde (PFA) for 5–10 min at room temperature and rinsed with water and stained with the acid phosphates, leukocyte (TRAP) kit (387A-1KT, Sigma, St. Louis, United States).

### Intracellular Acidification by Acridine Orange Staining

Intracellular acidification was determined by acridine orange (AO) fluorescence method. BMM-derived OCs or RAW264.7 cell-derived OCs were treated with Ad-GFP or Ad-Wnt7b for 12 h. Osteoclasts induced with 50 ng/mL RANKL for 5 days were incubated with 10 μg/mL AO for 15 min at 37°C. Cells were washed twice with PBS and processed for fluorescent microscopy analysis on a NIKON eclipse fluorescence microscope (Compix Inc., Sewickley, PA, United States) at excitation of 485 nm and emission of 520 nm. Images were performed using ImageJ (version 1.47).

### Adenovirus-Mediated Overexpression

To overexpress Wnt7b, RAW264.7 cells were infected with adenovirus encoding mouse Wnt7b or green fluorescent protein (GFP) purchased from Hanbio, Shanghai, China. For viral transfection, cells at the confluence of 80% were incubated with virus overnight, and medium was changed to complete medium. Quantitative PCR (qPCR) and fluorescent imaging were performed to verify transfection efficiency after 24 h transfection. Cells were used for further experiments upon verification.

### RNA Isolation and Quantitative PCR

Total RNA was extracted using TRIzol reagent (Invitrogen Inc., Carlsbad, CA, United States) and was reverse transcribed to cDNA with HiScript^®^ Q-RT SuperMix for qPCR (+ gDNA wiper) (Vazyme, Nanjing, China). Taq Pro Universal SYBR qPCR Master Mix was used for qPCR in CFX96 real-time system. Gene-specific primers are listed in Supplementary Table 1. Relative expression level was calculated by 2^–ΔΔCT^ and was normalized by β-actin gene.

### Western Blot

Cultured cells were washed with PBS and then lysed in radioimmunoprecipitation assay (RIPA) buffer containing protease and phosphatase inhibitor cocktail (Thermo Fisher Scientific, Hudson, NH, United States). Cell extracts were subjected to sodium dodecyl sulfate–polyacrylamide gel electrophoresis (SDS-PAGE) and Western blotting. Primary Abs used are listed in Supplementary Table 2. Horseradish peroxidase (HRP)-conjugated secondary antibodies were probed and developed with ECL solution. Signals were detected and analyzed by Bio-Rad ChemiDoc system (Bio-Rad, Hercules, CA, United States).

### Flow Cytometry Analysis and EdU Staining

Cells were cultured in complete medium for several hours at the confluence of 80%, and they were trypsinized, washed with PBS, and finally fixed in ice-cold 70% ethanol for at least 24 h. Cell cycle analyses followed manufacturer protocols (KGA512, KeyGEN Biotech, Nanjing, China). Cells were washed with PBS twice and incubated with RNase buffer for 30 min at 37°C and stained with PI solution for 30 min on ice. Cells were analyzed by guava easyCyte HT (Millipore, Billerica, MA, United States) and analyzed with software InCyte2.7 (Millipore, Billerica, MA, United States).

EdU staining was performed according to manufacturer’s guideline (C10310-1, Ribo Bio, Guangzhou, China). BMMs were cultured in complete medium at the confluence 50–70% and incubated with complete medium containing EdU at the concentration of 10 μM for 1 h. The labeled cells were subjected to EdU staining and counterstained with 4′,6-diamidino-2-phenylindole (DAPI). Images were captured by a NIKON eclipse fluorescence microscope (Compix Inc., Sewickley, PA, United States).

### Intracellular ATP Assays

Cells were cultured in 96-well plates and changed to 100 μL fresh complete medium before the assays. The CellTiter-Glo kit (G9241, Promega Corporation, Madison, WI, United States) was performed according to the manufacturer’s procedure, and luminescence of each well was detected from the opaque-welled 96-well plates. ATP levels were calculated according to the standard curve generated with 0.1, 1, and 10 μM of ATP and normalized to total DNA amount in each well.

Cell total DNA extraction method was as follows. Cultured cells were washed twice with Hank’s balanced salt solution (HBSS) (with CaCl_2_ and MgCl_2_, Gibco, Life Technologies Corporation, NY, United States). The plate was emptied before being frozen at −80°C for at least 1 h. About 100 μL or 1 mL of distilled water was added to each well of the plates and placed on an orbital shaker for 1 h at room temperature. One hundred microliters of cell lysates and 100 μL Hoechst 33342 (Thermo Fisher Scientific, Hudson, NH, United States) at 20 μg/mL in TNE buffer were mixed in a black 96-well plate, and fluorescence was detected. DNA amount was calculated according to the standard curve generated before.

### OVX Mice Model

Eight 8-week-old female wild-type mice and eight 8-week-old female *Wnt7b*^*LysM*^ mice were randomly divided into two groups to receive sham (four mice in this group) or bilateral OVX surgery (four mice in this group). After anesthesia by isoflurane and 100% oxygen for half hour, the mice in the OVX group were subjected to resection bilateral OVX, and the mice in the sham group underwent some fat tissue close to the ovaries, and all operations above were performed under aseptic conditions. Four weeks later, estrogen-deficiency−induced osteoporosis was successfully established by micro−CT scanning of the femurs of eight mice (four mice in each group) after sacrificed.

### Cell Proliferation Assay Analysis

Two groups (wild-type and Wnt7b^*LysM*^ group) of BMMs were cultured in six-well plates (Corning, Tewksbury MA, United States) at an initial density of 3.125 × 10^4^ cells/cm^2^. Cell proliferation was assessed using a water-soluble tetrazolium salt-WST-8 (Cell Counting Kit-8) according to the manufacturer’s instructions (DOJINDO, Tokyo, Japan). Then, CCK-8 data was read to obtain the absorbance at 450 nm, and the absorbance values of wild type were compared with those of Wnt7b*^*LysM*^* cells. Cell cycle was quantified by flow cytometry. Cells were stained with propidium iodide (PI) (KGA1015; KeyGEN Bio-TECH, Nanjing, China) following the manufacturer’s directions. Flow cytometry analysis was performed on more than 50,000 events. Data were analyzed with system software (Millipore Guava easyCyte HT, Merck Millipore, Darmstadt, Germany).

### Statistical Analysis

One-way ANOVA with *post hoc* Bonferroni correction was carried out for comparisons of multiple groups. Student’s *t*-test was performed to determine the statistical significance for two groups. The chi-square test was used to assess the portion of cells in different phases. The level of acceptable statistical significance was set at *p* < 0.05. Numerical data and histograms were presented as mean ± SD (standard deviations). Results were presented in the presence of at least three independent biological experiments, and for each individual experiment, at least three technical repeats were carried out. All fluorescent microscopic images were processed with Image Pro Plus 6.0 (Media Cybernetics, Rockville, MD, United States).

## Results

### Forced Expression of Wnt7b in Osteoclasts Enhances Bone Mass

We previously showed that mice with *DMP1*-driven *Wnt7b* overexpression exhibited a high bone mass phenotype. Moreover, the osteogenic differentiation of BMSCs was significantly promoted as expected, which indicated that Wnt7b is a potent activator for osteogenesis (Yu et al., 2020). The reduction in osteoclasts in mutant mice inspired us that Wnt7b might play an important role in osteoclastogenesis in either a cell autonomous or non-autonomous manner.

**FIGURE 1:**
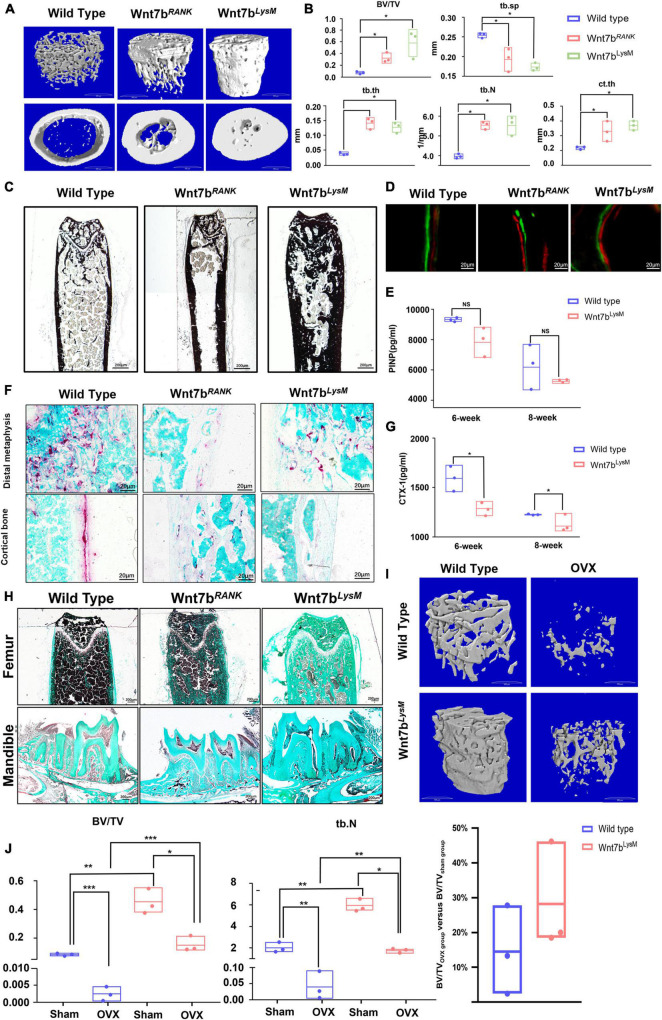
Wnt7b-specific overexpression in osteoclast precursors presents a high bone mass phenotype and a positive outcome in ovariectomy-induced bone loss. **(A)** 3D microcomputer tomography (μCT) reconstruction image of femoral cortical bones and trabecular bones from 3-month-old mice. **(B)** Quantification of the trabecular bone and cortical bone parameters in the femur. *N* = 3. **(C)** Van Kossa staining of the femurs from *WT* mice, *Wnt7b*^*RANK*^ mice and *Wnt7b*^*LysM*^ mice. Van Kossa staining was executed with non-decalcified frozen sections. Representative images were shown. **(D)** Representative images of Calcein-Alizarin double labeling in femurs. **(E)** Quantitative analysis of serum bone-formation marker PINP. **(F)** TRAP staining of distal metaphysis and diaphyseal cortical bone of the femur from *WT* mice, *Wnt7b*^*RANK*^ mice, and *Wnt7b*^*LysM*^ mice. **(G)** Quantitative analysis of serum bone-resorption marker CTX-1. **(H)** Goldner’s Trichrome staining of the femurs and mandibles from *WT* mice, *Wnt7b*^*RANK*^ mice, and *Wnt7b*^*LysM*^ mice. Samples were collected from 3-month-old mice. Goldner Trichrome staining was performed following the manufacturer’s protocol (Goldner Trichrome stain kit, Solarbio, Beijing, G3550). **(I)** Reconstruction 3D image and **(J)** quantification parameters of trabecular bone from *WT* mice and *Wnt7b*^*LysM*^ mice established OVX model or not. *p < 0.05; **p < 0.01; ***p < 0.001.

To test this, we planned to specifically activate Wnt7b in osteoclasts. To this end, we generated *RANK-Cre;Rosa26^*Wnt*7b/+^* (hereafter referred to as *Wnt7b*^*RANK*^) and *LysM-Cre;Rosa26^*Wnt*7b/+^* (hereafter referred to as *Wnt7b*^*LysM*^) conditional knockout mice by crossing *Rosa26-Wnt7b* with either *RANK-Cre* or *LysM-Cre* transgenic mice. At 12 weeks of age, microcomputed tomography (μCT) scanning of the proximal femora was performed. Interestingly, similar to the overexpression of Wnt7b in osteoblasts, the three-dimensional reconstruction of μCT scanning showed a significant increase in bone mass in both trabecular and cortical bone in *Wnt7b*^*RANK*^ and *Wnt7b*^*LysM*^ mice compared to their littermates (*Rosa26^*Wnt*7b^*, hereafter referred to as *wildtype or WT*) (Figure 1A). The μCT analysis revealed that the trabecular bone mass (BV/TV) was increased in *Wnt7b*^*RANK*^ and in *Wnt7b*^*LysM*^ compared to *WT*. The increase in bone mass in mutant mice was due to higher trabecular number (Tb.N.), higher trabecular thickness (Tb.Th), and a decrease in trabecular spacing (Tb.Sp.). The μCT analysis also showed a thicker cortical bone diameter in mutant mice indicated by elevated cortical bone thickness (Ct.Th.) (Figure 1B). Von Kossa staining and Goldner trichrome staining demonstrated deeper staining and thicker bone in mutant mice compared to *WT*, suggesting more calcium deposition (Figure 1C). Consistent with the lack of change in new bone formation revealed by dynamic histomorphometry in the femur, the serum marker PINP was not impaired (Figures 1D,E). However, we observed a reduced number of TRAP-positive cells in the trabeculae (Figure 1F) and a significantly decreased bone resorption serum marker CTX-1 in mutant mice compared to *WT* (Figure 1G). The number of TRAP-positive cells in the mandible was diminished in mutant mice (Figure 1H). These data suggest that the specific gain-of-function of *Wnt7b* in osteoclasts significantly increased bone mass due to a defect in osteoclast differentiation and activity *in vivo*.

### Wnt7b Increases Bone Mass in Ovariectomized Mice

According to the above results, we suggest that Wnt7b has the potential to relieve pathological bone resorption. To further examine this possibility, we generated an OVX mouse model, which exhibited intense bone resorption caused by an estrogen deficit. Six weeks after ovariectomy, μCT analysis revealed that Wnt7b overexpression increased bone mass in both sham and OVX group (Figure 1I). Although we found that the ratio of BV/TV with Wnt7b overexpression showed an increasing trend, the difference was not statistically significant. Due to the lack of changes in the level of reduction in BV/TV, we concluded that Wnt7b achieved a beneficial effect on bone mass, rather than playing a protective effect on OVX-induced osteoporosis *in vivo* (Figures 1I,J).

### Wnt7b Impairs Osteoclast Formation *in vitro*

**FIGURE 2:**
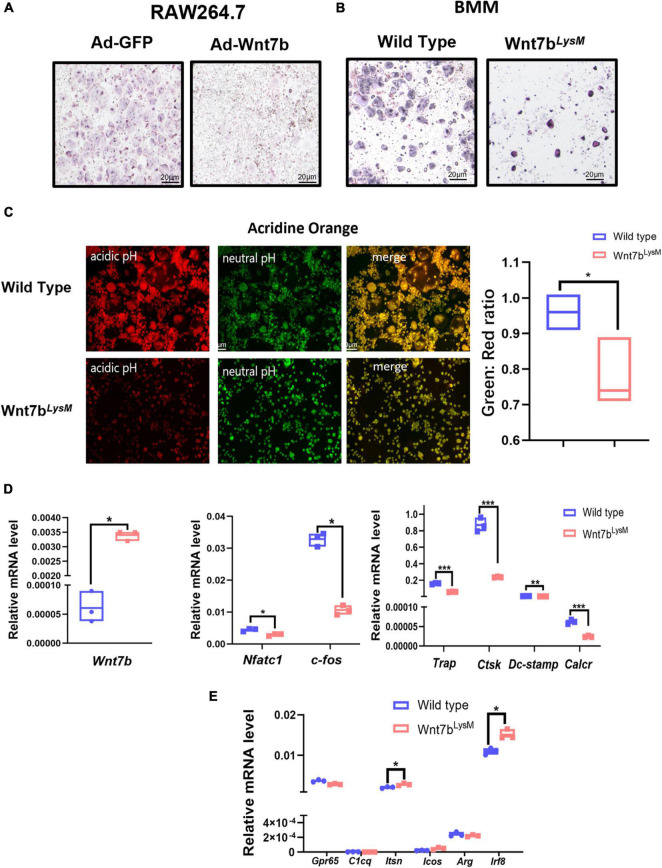
Wnt7b impairs osteoclastogenesis in BMMs. **(A)** Raw264.7 transfected with adeno-GFP or adeno-Wnt7b and **(B)** BMMs isolated from *WT* or *Wnt7b*^*LysM*^ mice were induced with M-CSF and RANKL for 5 days. **(A,B)** TRAP staining was used to identify TRAP-positive multinucleated cells. **(C)** Acridine orange (AO) staining was applied to access the acidification of RANKL-induced multinucleated cells at day 5. Green represents neutral pH, and red represents acidic pH. Intracellular pH at day 5 is decreased after in the presence of Wnt7b compared to wild type. **(D)** The expression levels of genes including early osteoclastic differentiation markers and osteoclast functional markers were detected by real-time PCR analysis. **(E)** Negative regulators in BMMs without RANKL inducement were screened by quantitative PCR. *N* = 3. *p < 0.05; **p < 0.01; ***p < 0.001.

*In vitro*, the BMMs from *Wnt7b*^*LysM*^ mice under RANKL induction formed less osteoclast than those from *WT* mice as indicated by TRAP staining. Besides, Wnt7b overexpression in RAW264.7 cells and BMMs also abolished osteoclastogenesis (Figures 2A,B). We next performed acridine orange staining on osteoclasts to visualize the intracellular acidification (Figure 2C). The osteoclasts differentiated from BMMs from *Wnt7b*^*LysM*^ mice had less acidification than those from *WT* mice in the presence of RANKL, which indicated a reduced osteoclast activity. Besides, qPCR experiments indicated that mRNA expression of osteoclast-specific genes, including *Nfatc1*, *C-fos*, *Trap*, *Ctsk*, *Dc-stamp*, and *Calcr*, were significantly repressed in the presence of Wnt7b under osteoclastogenic induction (Figure 2D). Of note, the negatively regulated genes during osteoclast differentiation were also screened by qPCR. Among these genes, *Irf8* and *Itsn* were increased due to *Wnt7b* overexpression in BMMs (Figure 2E). These results demonstrate that Wnt7b has inhibitory effects on osteoclast differentiation and maturation *in vitro*.

### Wnt7b Suppresses β-Catenin-Dependent Signal in Bone Marrow Macrophages

**FIGURE 3:**
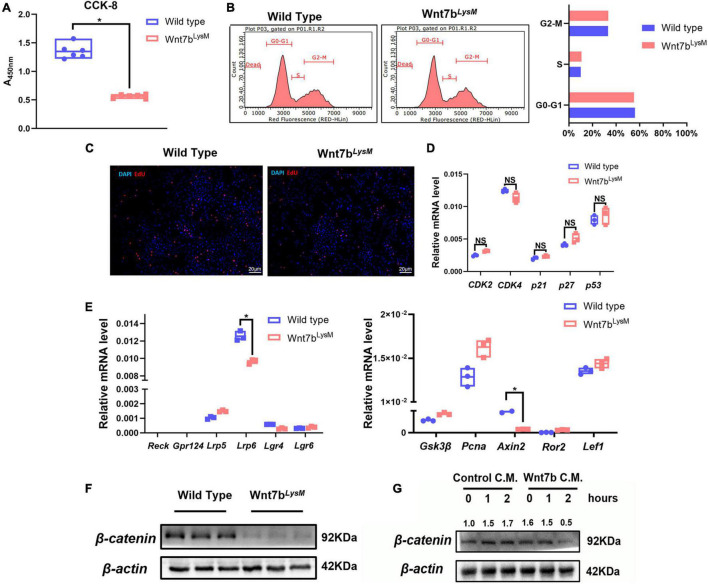
Wnt7b suppressed canonical Wnt pathway in BMMs. **(A)** Cell Count Kit-8 (CCK-8) of BMMs derived from *WT* or *Wnt7b*^*LysM*^ mice was performed to assessed cell viability. **(B)** Cell cycle distribution detected by flow cytometry analysis. **(C)** Representative images of 5-ehtynyl-2′-deoxyuridine (EdU) staining of BMMs derived from *WT* or *Wnt7b*^*LysM*^ mice. **(D)** Transcriptional levels of cell-cycle-related genes (*CDK2*, *CDK4*, *p21*, *p27*, and *p53*) determined by real-time PCR. **(E)** Expression levels of genes including Wnt receptors (*Reck*, *Gpr124*, *Lrp5/6*) and Wnt-related genes (*Gsk3*β, *Axin2*, *Ror2*, *Lef1*, and *Pcna*) were determined by real-time PCR. **(F)** Protein level of β-catenin in BMMs isolated from *WT* or *Wnt7b*^*LysM*^ mice. **(G)** Raw264.7 cells were treated with conditional medium from 293FT cell line transfected with adeno-GFP or adeno-Wnt7b for the indicated period. Whole-cell lysates were collected for Western blot detection for β-catenin. The relative densities of blots are shown on top. *N* = 3. *p < 0.05; NS: not significant.

Previous studies have demonstrated that Wnt ligands such as Wnt3a can modulate the proliferation of BMMs through the canonical Wnt pathway and that β-catenin is essential for osteoclast precursors (Hamamura et al., 2014; Weivoda et al., 2016). Therefore, we next examined the cell viability of BMMs in the presence of Wnt7b using CCK-8 cell counting experiments. As a result, we detected a reduction in the viability of BMMs derived from *Wnt7b*^*LysM*^ mice indicated by CCK-8 experiment (Figure 3A). Next, flow cytometry was used to analyze the phases of the cell cycle. Surprisingly, there was no remarkable difference between *Wnt7b* overexpression and control in terms of cell cycle (Figure 3B). EdU staining of cultured BMMs from either *WT* or *Wnt7b*^*LysM*^ mice also confirmed the flow cytometry results (Figure 3C). Meanwhile, the cell-cycle-related genes, such as *Cdk2*, *Cdk4*, *P21*, *P27*, and *P53*, did not change when enforced Wnt7b expression in BMMs (Figure 3D). Next, mRNA expression of Wnt receptors, such as *Reck*, *Gpr124*, and *Lrp5/6*, and Wnt downstream genes, such as *Axin2* and *Gsk3*β, *Lef1*, *Ror2*, and *Pcna*, were examined by qPCR (Figure 3E). We found that the mRNA expression of majority of Wnt receptors showed no significant changes between WT and *Wnt7b*^*LysM*^, and genes related to β-catenin-dependent Wnt signal did not activate in response to Wnt7b in BMMs. Furthermore, the decreased transcriptional level of Lrp6 and Axin2 in the presence of Wnt7b indicated that the canonical Wnt signal might be suppressed in BMMs with Wnt7b overexpression. We further explored the protein levels of β-catenin in cell lysates by Western blotting and found that β-catenin was obviously decreased in the BMMs derived from *Wnt7b*^*LysM*^ mice compared to those from the *WT* mice (Figure 3F). In addition, we treated the BMMs with Wnt7b conditional medium and detected that the protein level of β-catenin in BMMs was also reduced in response to Wnt7b in a time-dependent manner (Figure 3G). Data indicated that the β-catenin-dependent pathway was suppressed in the presence of Wnt7b in BMMs.

### Wnt7b Inhibits Osteoclastogenesis by Suppressing the AKT Signaling Pathway

**FIGURE 4:**
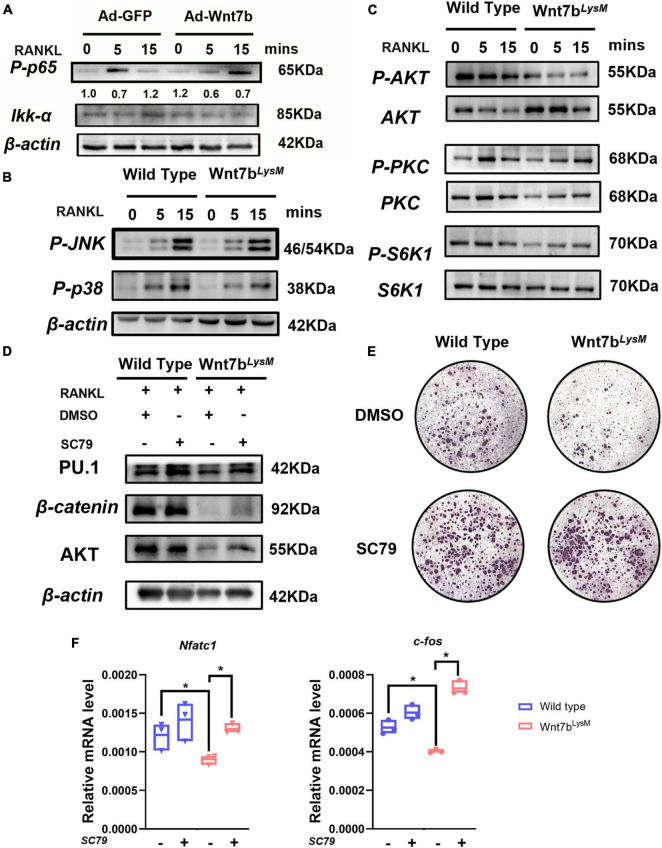
Wnt7b inhibited osteoclast differentiation by suppressing AKT phosphorylation. **(A)** Raw264.7 cell line transfected with adeno-GFP as control or adeno-Wnt7b was treated with RANKL for the indicated periods. Cell lysates were subjected to Western blot (WB) analysis for canonical NF-κB signal pathway. The relative densities of blots are shown on top of IKK-a. **(B,C)** BMMs isolated from *WT* or *Wnt7b*^*LysM*^ mice were stimulated with RANKL for increasing time. Whole-cell lysates were collected for Western blotting analysis of targets of MAPK pathway (p-p38 and p-JNK) and targets of mTOR pathway (p-AKT, AKT, p-PKC, PKC, p-S6K1, and S6K1). **(D)** BMMs obtained from *WT* or *Wnt7b*^*LysM*^ mice were incubated with RANKL for 1 day together with vehicle (DMSO) or SC79 addition. The expression levels of early osteoclast differentiation proteins in BMMs were analyzed by WB. **(E)** Osteoclastogenesis were determined with RANKL for 5 days with SC79 or DMSO. TRAP staining was performed to identify the TRAP-positive multinucleated cells. **(F)** The expression levels of early osteoclast differentiation markers in BMMs were analyzed by quantitative PCR after treated with SC79 or DMSO. *N* = 3. *p < 0.05.

Several signaling pathways, such as nuclear factor kappa B (NF-κB), mitogen-activated protein kinase (MAPK), and mammalian target of rapamycin (mTOR), are involved in osteoclast differentiation (Liu and Zhang, 2015). We screened whether Wnt7b inhibited the key activation step of several signaling pathways induced by RANKL. In the canonical NF-κB pathway, phosphorylation and nuclear translocation of p65 plays a critical role during RANKL-induced NF-κB activation. Western blotting analysis revealed that Wnt7b inhibited or at least delayed RANKL-induced p65 phosphorylation (Figure 4A) and the non-canonical NF-κb activation indicated by Ikk-a protein level (Abu-Amer, 2013). The RANKL-induced p38 and JNK phosphorylation also was not affected by Wnt7b overexpression (Figure 4B). Since AKT, a critical serine-threonine kinase, is reportedly mediating diverse signaling pathway downstream of mTORC2, we tested the phosphorylation of AKT at Ser473. We observed that Wnt7b significantly abolished AKT phosphorylation at Ser473, whereas activation of PKC and S6K1 was not significantly impaired with RANKL treatment for 15 min (Figure 4C).

Next, we investigated whether SC79, an AKT activator, rescued osteoclast formation suppressed by Wnt7b (Zhang et al., 2016). We pretreated BMMs with SC79 or dimethyl sulfoxide (DMSO) for 24 h before inducing them with RANKL for additional 5 days. The protein expression of osteoclast-differentiation-related genes were upregulated after SC79 treatment (Figure 4D). Of note, BMMs derived from *Wnt7b*^*Lysm*^ mice previously failed to form TRAP-positive cells, whereas the number of osteoclasts increased with SC79 treatment in the presence or absence of Wnt7b (Figure 4E). The mRNA expressions of osteoclast-differentiation-related genes were rescued after SC79 treatment (Figure 4F). Thus, we concluded that Wnt7b abolished osteoclast formation by inhibiting AKT phosphorylation.

### Wnt7b Rewires the Metabolic Process in Bone Marrow Macrophages

**FIGURE 5:**
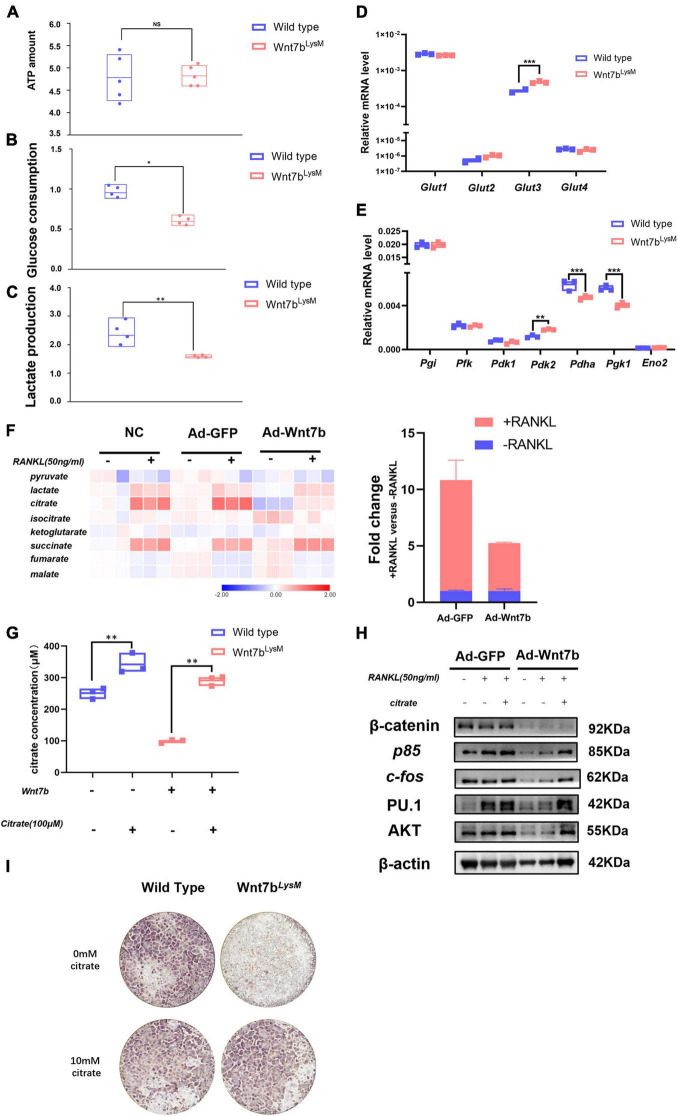
Wnt7b rewires glucose metabolic process in BMMs through regulating AKT degradation. **(A–C)** Total ATP amount, glucose consumption, and lactate production of Raw264.7 infected with adeno-GFP or adeno-Wnt7b were determined. **(D,E)** The expression levels of genes of Glut family (*Glut1*, *Glut2*, *Glut3*, *Glut4*) and glycolysis-related enzymes were analyzed by real-time PCR in primary BMMs. **(F)** Metabolic profile of intermediates in TCA cycle in Raw264.7 cells infected with adeno-GFP or adeno-Wnt7b in the presence or absence of RANKL. The fold changes of citrate are shown to the right. **(G)** Intracellular citrate amount in Raw264.7 cells after adeno-GFP or adeno-wnt7b transfection in the presence or absence of exogenous citrate. **(H)** Raw264.7 cells after adeno-GFP or adeno-Wnt7b transfection were induced with RANKL plus citrate for 1 day. Western blot was used to determine the protein levels of β-catenin, p85, AKT, c-fos and P.U 1. **(I)** TRAP staining was used to identify TRAP-positive multinucleated cells. BMMs isolated from *WT* or *Wnt7b*^*LysM*^ mice were induced with M-CSF and RANKL for 5 days in addition with or without 10 mM citrate; then, TRAP staining was performed as described. Each dot represented one sample. NS: not significant; *p < 0.05; **p < 0.01; ***p < 0.001.

Recent studies have shown that the cell metabolic status could regulate signal pathway activation and contribute to the regulation of cell differentiation (Lecka-Czernik and Rosen, 2015). Moreover, ATP generated from anerobic glycolysis has been shown to fuel the PI3K-AKT pathway to support the Th17 cell response (Xu et al., 2021a,b). Previous studies have revealed that glycolysis and OXPHOS were both enhanced during osteoclast differentiation. Meanwhile, glucose consumption and the expression of glucose transporters (GLUTs) were elevated as the osteoclast differentiated (Li et al., 2020). Therefore, we examined ATP levels, glucose consumption, and lactate production to determine the metabolic condition of BMMs derived from *Wnt7b*^*Lysm*^ mice compared to those from *WT* mice (Figures 5A–C). The ATP levels were unchanged; however, glucose consumption and lactate production dropped significantly. Then, we screened the transcriptional level of GLUTs family in BMMs. Glut1, a member of glucose transporter family, identified as the predominant GLUTs in BMMs, showed no significant change in the presence of Wnt7b, while Glut3 presented a mild increase (Figure 5D). Thus, GLUT family was dispensable for the inhibition of glucose utilization caused by Wnt7b.

Furthermore, the transcriptional levels of the metabolic enzymes were examined by qPCR. The data demonstrated that most of the glycolysis-related genes were steadily expressed, whereas the expression of *Pgk1* and *Pdha* was dramatically decreased in the presence of Wnt7b (Figure 5E). Some published studies revealed that *Pgk1* was a central enzyme in glycolysis process, which generated a molecular of ATP. The mRNA expression of Pgk1 decreased in BMMs with Wnt7b forced expression suggesting that Wnt7b impaired glycolysis in BMMs with Wnt7b overexpression. Meanwhile, we found that Wnt7b forced expression led to slight upregulation of Pdk2 and downregulation of *Pdha*, which controlled the turnover of TCA cycle. These data suggested that the glucose metabolic process was rewired by Wnt7b in BMMs.

Next, we assayed the metabolites in BMMs with or without *Wnt7b* overexpression for 24 h before RANKL treatment (Figure 5F). The metabolites in the TCA cycle were elevated in RANKL-stimulated BMMs compared to the unstimulated BMMs, which indicated an increasing glucose usage in TCA during osteoclastogenesis. Importantly, with RANKL inducement, the content of citrate was significantly reduced in the presence of Wnt7b. Therefore, we investigated whether resupplemented citrate into the medium rescued osteoclast formation, which was suppressed by Wnt7b. To this end, we first tested the intracellular amount of citrate in BMMs after citrate compensation. After treatment with additional citrate in the medium, the intracellular citrate content was increased in BMMs (Figure 5G), as were the protein levels of AKT and the osteoclast differentiation markers PU.1 and C-fos (Figure 5H). Moreover, the TRAP staining also demonstrated that the BMMs derived from *Wnt7b*^*LysM*^ mice form more osteoclasts in the presence of 10 mM citrate (Figure 5I), which indicated that citrate compensation rescued osteoclastogenesis. Therefore, we concluded that Wnt7b impaired AKT phosphorylation and rewired the glucose metabolic process in BMMs during osteoclastogenesis.

## Discussion

Over the past decades, the Wnt signaling pathway has been demonstrated as a well-established regulator of skeleton homeostasis (Baron and Kneissel, 2013; Huybrechts et al., 2020). Several reports have shown that Wnt ligands have a dual effect on bone mass, which affects both osteoblasts and osteoclasts (Glass et al., 2005). In recent years, emerging evidence has indicated that the Wnt pathway plays an indispensable role in osteoclastogenesis (Kobayashi et al., 2019). For example, forced expression of the dominant active form of β-catenin in osteoblasts leads to the development of a high bone mass phenotype with impaired osteoclast differentiation (Glass et al., 2005). The biphasic regulatory effect of the Wnt ligand Wnt3a on osteoclastogenesis is dependent on β-catenin. Wnt3a enhances the proliferation of osteoclast precursors through the activation of β-catenin and impairs the differentiation of osteoclast precursors due to β-catenin constitutive activation (Sui et al., 2018). Besides, the stimulation of OPG in osteoblasts can also block osteoclastogenesis. Wnt16, another Wnt ligand, has been reported to inhibit osteoclast differentiation through the non-canonical Wnt pathway (Mizoguchi et al., 2009; Movérare-Skrtic et al., 2014). During the inhibition, Wnt16 fails to induce the accumulation of β-catenin or expression of Axin2 in osteoclast precursors; however, it is able to decrease the expression of NFATc1 in these precursors (Mizoguchi et al., 2009). Besides, the NF-κB signal, a crucial signaling pathway for osteoclastogenesis, is also suppressed in osteoclast progenitors following Wnt16 treatment. Furthermore, in *in vitro* cell culture, Wnt16 can induce OPG expression, which protects the skeleton from excessive bone resorption and blocks osteoclast differentiation by binding to RANKL (Movérare-Skrtic et al., 2014). Therefore, these findings suggest that Wnt16 negatively regulates osteoclast formation *via* both direct and indirect mechanisms. Wnt4, another inhibitory Wnt for osteoclastogenesis, impairs osteoclast formation in a β-catenin-independent manner (Yu et al., 2014). Osteoclast precursors fail to differentiate into osteoclasts following treatment with Wnt4 due to suppression of NF-κB activation. Consequently, the expression of NFATc1 in these cells is inhibited by Wnt4. Wnt4 does not inhibit RANKL-induced osteoclast differentiation but blocks 1α,25(OH)_2_D3-induced osteoclast formation when co-cultured with osteoblasts. These results demonstrate that Wnt4 inhibits osteoclast formation through the interaction with osteoblasts. As a non-canonical WNT ligand, Wnt5a binding to Ror1 or Ror2 regulates cell polarity and invasiveness through the β-catenin-independent pathway (Maeda et al., 2012). Previous studies have demonstrated that Wnt5a derived from osteoblasts promotes osteoclastogeneis. Mechanistically, Wnt5a enhances the expression of RANK by activating c-Jun in the osteoclast precursors, thereby promoting RANKL-induced osteoclastogenesis (Ikeda et al., 2004). In addition, Wnt5a also facilitates Wnt/β-catenin signals by promoting the expression of Lrp5/6, which further reduces osteoclast formation. Thus, Wnt ligands can regulate osteoclast differentiation in both direct and indirect manners.

Taken together, these results indicate that Wnt7b has potent osteogenic activity (Chen et al., 2014, 2019; Yu et al., 2020). Specific overexpression of Wnt7b in osteoblasts markedly increased the number and function of osteoblasts. Further investigation of the molecular mechanisms suggested that Wnt7b promotes bone formation through multiple signals. mTORC1 was activated by Wnt7b in osteoblasts through the PI3k-AKT pathway instead of the canonical Wnt signaling pathway. Recent studies have uncovered the link between glucose metabolism and bone formation. Wnt7b has been shown to enhance glycolysis in osteoblasts through increasing Glut1 expression. In addition to the potent bone anabolic effect, Wnt7b appears to abolish osteoclast numbers. Moreover, overexpression of Wnt7b in DMP1-Cre mice showed a high bone mass phenotype and suppression of osteoclast numbers.

Our results showed that Wnt7b directly acts on osteoclast precursors through the β-catenin-independent pathway. Wnt7b had no significant effect on the cell cycle of osteoclast precursors Wnt7b but did inhibit osteoclast differentiation by suppressing AKT phosphorylation during RANKL induction. Akt activation was crucial for NF-κB signals and NFATc1 expression in response to RANKL in osteoclast progenitors (Tiedemann et al., 2017; Fu et al., 2020; Xin et al., 2020). We found that the inhibitory effect of Wnt7b was reversed by the Akt-specific activator SC79, and at the same time, the Wnt7b-mediated downregulated expression of NFATc1 was restored with SC79 treatment.

Recent studies have shown that the phosphorylation of AKT depends on glycolysis and is controlled by several metabolic intermediators (Lecka-Czernik and Rosen, 2015; Covarrubias et al., 2016; Martinez Calejman et al., 2020; Xu et al., 2021a). Wnt7b suppressed the glucose consumption and lactate production in osteoclast precursors and decreased the amount of citrate in these cells. As previously reported, additional low concentration of pyruvate or citrate augments the osteoclasts formation by enhancing cell energy metabolism, and the amount of citrate was significantly raised during RANKL-induced osteoclast differentiation (Fong et al., 2013). Citrate, a well-known TCA intermediator, can act as a signaling molecule to trigger some physiological response. Citrate induced upregulation and phosphorylation of AKT in endothelial cells and some tumor cells to enhance cancer cells invasion and metastasis. However, several studies indicated citrate suppressed tumor growth through activation of PTEN (Ren et al., 2017). We found that Wnt7b impaired AKT phosphorylation and decreased citrate content in TCA cycle, which indicated a glucose metabolic switch in the presence of Wnt7b. We supplemented BMMs culture medium with 10 mM citrate and found that the amount of AKT in Wnt7b-treated cells was rescued. All these results reflected that Wnt7b suppressed osteoclasts formation *via* AKT- and citrate-dependent pathways. However, we still cannot address the interaction between citrate and AKT currently.

## Conclusion

In conclusion, our study provides evidence to demonstrate that Wnt7b increases bone mineral density and improves bone biochemical properties in mice by inhibiting bone resorption through β-catenin-independent pathways. The potential therapeutic use of Wnt7b as a novel bone formation and bone resorption dual regulator in postmenopausal osteoporosis is worthy of further investigation.

## Data Availability Statement

The original contributions presented in the study are included in the article/Supplementary Material, further inquiries can be directed to the corresponding author/s.

## Ethics Statement

The animal study was reviewed and approved by the Ethical Committees of the State Key Laboratory of Oral Diseases, Sichuan University.

## Author Contributions

FW, BL, and XH conducted the experiments and acquired the data. FW and FY analyzed the data. YS and LY helped with critical advices and discussion. LY designed the project and oversaw the project and revised the manuscript. FW and YS drafted the manuscript. All authors reviewed the manuscript.

## Conflict of Interest

The authors declare that the research was conducted in the absence of any commercial or financial relationships that could be construed as a potential conflict of interest.

## Publisher’s Note

All claims expressed in this article are solely those of the authors and do not necessarily represent those of their affiliated organizations, or those of the publisher, the editors and the reviewers. Any product that may be evaluated in this article, or claim that may be made by its manufacturer, is not guaranteed or endorsed by the publisher.
