# Supplementary material for: Wnt7b Inhibits Osteoclastogenesis via AKT Activation and Glucose Metabolic Rewiring
Source: Front Cell Dev Biol. 2021 Nov 22;9:771336. doi: 10.3389/fcell.2021.771336 (PMC8645835; doi:10.3389/fcell.2021.771336)
Supplement: Supplementary file 1 [file Data_Sheet_1.docx]

Table S1 Primers for quantitative PCR.

| **Gene** | **Sequence** |
| --- | --- |
| **Cdk2_Mm_F** | **GCATTCCTCTTCCCCTCATC** |
| **Cdk2_Mm_R** | **GGACCCCTCTGCATTGATAAG** |
| **Cdk4_Mm_F** | **ACAAGTAATGGGACCGTCAAG** |
| **Cdk4_Mm_R** | **GGGTGTTGCGTATGTAGACTG** |
| **p21_Mm_F** | **CTTGCACTCTGGTGTCTGAG** |
| **p21_Mm_R** | **GCACTTCAGGGTTTTCTCTTG** |
| **p53_Mm_F** | **ATGTTCCGGGAGCTGAATG** |
| **p53_Mm_R** | **CCCCACTTTCTTGACCATTG** |
| **p27_Mm_F** | **TGGACCAAATGCCTGACTC** |
| **p27_Mm_R** | **GGGAACCGTCTGAAACATTTTC** |
| **Acly_Mm_F** | **AAGCCTACATTGCAGACCTG** |
| **Acly_Mm_R** | **TTGACACCTCCAAGATCACAG** |
| **CS_Mm_F** | **GGGACTTGTGTATGAGACTTCG** |
| **CS_Mm_R** | **AGCCAAAATAAGCCCTCAGG** |
| **Glut1_Mm_F** | **GATTGGTTCCTTCTCTGTCGG** |
| **Glut1_Mm_R** | **CCCAGGATCAGCATCTCAAAG** |
| **Glut2_Mm_F** | **GTCACTATGCTCTGGTCTCTG** |
| **Glut2_Mm_R** | **CAAGAGGGCTCCAGTCAATG** |
| **Glut3_Mm_F** | **CGCTTCTCATCTCCATTGTCC** |
| **Glut3_Mm_R** | **TGAAGATAGTATTGACCACGCC** |
| **Glut4_Mm_F** | **GTTGGTCTCGGTGCTCTTAG** |
| **Glut4_Mm_R** | **ACATAGCTCATGGCTGGAAC** |
| **Pdk1_Mm_F** | **GACTGTGAAGATGAGTGACCG** |
| **Pdk1_Mm_R** | **CAATCCGTAACCAAACCCAG** |
| **Pdk2_Mm_F** | **AAGAGATCAACCTGCTTCCTG** |
| **Pdk2_Mm_R** | **GCATCTGTGAACTGGCTTAGAG** |
| **Pdha_Mm_F** | **CAGAGCTAACAGGACGAAGAG** |
| **Pdha_Mm_R** | **TCCATTGTACTTGCAGGCC** |
| **Idh1_Mm_F** | **TGGCCTTTGTATCTCAGCAC** |
| **Idh1_Mm_R** | **TGAGCCTGTGTTCATAGCAG** |
| **Idh2_Mm_F** | **CAAGGAGTGGGAGGTGTATAAC** |
| **Idh2_Mm_R** | **TCAAGTAGAGCGGCCATTTC** |
| **c-fos_Mm_F** | **CAAGCGGAGACAGATCAACTTG** |
| **c-fos_Mm_R** | **TTTCCTTCTCTTTCAGCAGATTGG** |
| **DC-Stamp_Mm_F** | **ACAAACAGTTCCAAAGCTTGC** |
| **DC-Stamp_Mm_R** | **TCCTTGGGTTCCTTGCTTC** |
| **Trap_Mm_F** | **AAGCGCAAACGGTAGTAAGG** |
| **Trap_Mm_R** | **CGTCTCTGCACAGATTGCAT** |
| **Calcr_Mm_F** | **ATCAGTTGCCCTCTTATGAAGG** |
| **Calcr_Mm_R** | **GTGTCAAAGTCCGGGAAGTAG** |
| **Ctsk_Mm_F** | **AGGCAGCTAAATGCAGAGGGTACA** |
| **Ctsk_Mm_R** | **AGCTTGCATCGATGGACACAGAGA** |
| **Nfatc1_Mm_F** | **GGTAACTCTGTCTTTCTAACCTTAAGCTC** |
| **Nfatc1_Mm_R** | **GTGATGACCCCAGCATGCACCAGTCACA** |
| **Lrp5_Mm_F** | **ACTCCAGCTTCACTCCGC** |
| **Lrp5_Mm_R** | **CTGTACTGCAGCTTGGTCCC** |
| **Lrp6_Mm_F** | **CCTCCAAGCCTCCAACTACA** |
| **Lrp6_Mm_R** | **CTTCTGCGTGCTGCTGAG** |
| **Lgr4_Mm_F** | **AAGATAACAGCCCCCAAGAC** |
| **Lgr4_Mm_R** | **AGGCAGTGATGAACAAGACG** |
| **Lgr6_Mm_F** | **ACATAACAACCGCATCCAGC** |
| **Lgr6_Mm_R** | **ATGCTGACCTTCCCACAAAC** |
| **Gsk3β_Mm_F** | **TCCATTCCTTTGGAATCTGC** |
| **Gsk3β_Mm_R** | **CAATTCAGCCAACACACACAGC** |
| **Pcna_Mm_F** | **GGTCTCGGCATATACGTGCAA** |
| **Pcna_Mm_R** | **AGCAACTTGGAATCCCAGAACA** |
| **Axin2_Mm_F** | **GGTTCCGGCTATGTCTTTGC** |
| **Axin2_Mm_R** | **CAGTGCGTCGCTGGATAACTC** |
| **Lef1_Mm_F** | **CCCTCCTACTCCAGTTACTCT** |
| **Lef1_Mm_R** | **CCACGGGCACTTTATTTGAT** |
| **Irf8_Mm_R** | **GGAAAGCCTTACCTGCTGAC** |
| **Irf8_Mm_F** | **AAGGTCACCGTGGTCCTTAG** |
| **Arhgap15_Mm_R** | **GTGTCTTTCAGGTAAGAATGCCA** |
| **Arhgap15_Mm_F** | **GCTCAGATTTGGAGGGAAAAGGA** |
| **C1qc_Mm_R** | **CCCAGTTGCCAGCCTCAAT** |
| **C1qc_Mm_F** | **GGAGTCCATCATGCCCGTC** |
| **Gpr65_Mm_R** | **ATGGCGATGAACAGCATGTG** |
| **Gpr65_Mm_F** | **ACGCATAAAGATCCGATGTTGG** |
| **Icosl_Mm_R** | **TAAAGTGTCCCTGTTTTGTGTCC** |
| **Icosl_Mm_F** | **ATTGCACCGACTTCAGTCTCT** |
| **Itsn1_Mm_R** | **CTGAAGCCGATAGCGGGATTT** |
| **Itsn1_Mm_F** | **GGCTGAGGTAACCCAGATTGG** |

Table S2 The Primary antibodies for Western Blot.

| **Pathways** | **Antibody** | **Cat No.** |
| --- | --- | --- |
|  | **NF-κB1** | **ZEN-BIOSCIENCE 200965** |
|  | **NF-κB2** | **ZEN-BIOSCIENCE 861024** |
| **NF-κB** | **P-p65** | **ZEN-BIOSCIENCE 380738** |
|  | **RelB** | **ZEN-BIOSCIENCE 381322** |
|  | **c-Rel** | **ZEN-BIOSCIENCE 122147** |
|  | **S6K1** | **ZEN-BIOSCIENCE 380469** |
|  | **P-S6K1** | **ZEN-BIOSCIENCE 380880** |
| **mTOR** | **AKT** | **ZEN-BIOSCIENCE 342529** |
|  | **P-AKT(S473)** | **ZEN-BIOSCIENCE 310021** |
|  | **P-PKC** | **ZEN-BIOSCIENCE R22939** |
|  | **PKC** | **ZEN-BIOSCIENCE 385382** |
|  | **P-p38** | **ZEN-BIOSCIENCE 310068** |
|  | **p38** | **ZEN-BIOSCIENCE 200782** |
| **MAPK** | **P-JNK** | **ZEN-BIOSCIENCE 340778** |
|  | **JNK** | **ZEN-BIOSCIENCE 380556** |
|  | **β-catenin** | **ZEN-BIOSCIENCE 250110** |
|  | **HRP-β-actin** | **Proteintech HRP-60008** |
